# The National Association of Dental Technics

**Published:** 1895-09

**Authors:** 


### The National Association of Dental Technics.

The second annual meeting of this body was held at Asbury Park, August 6th, 7th and 8th, 1895. President D. M. Cattell, in the Chair. This organization has for its special object the promotion and elevation of demonstrative teaching in our colleges, and a more important subject could hardly be presented; for, notwithstanding the great progress that has been made in this direction in our educational institutions, vastly more can, and will be done, by combined effort such as is now being put forth. It is practically making the advance, that may be made by any one, the common property of all; and, indeed, in this matter bringing all to a common level, and to the possession of the best methods.

With such combined effort in operation it is impossible to estimate the possibilities of the future of dentistry.

The student of today has very little appreciation of the advantages within his reach, when compared with the former times, and even with those of a little while ago.

The officers for the present year are as follows: President, Dr. T. E. Weeks, of Minneapolis; Vice-President, Dr. S. H. Guilford, of Philadelphia; Secretary-Treasurer, Dr. J. T. Stephan, of Cleveland; Executive Board, Dr. D. M. Cattell, of Chicago, Dr. N. S. Hoff, of Ann Arbor and Dr. H. W. Morgan, of Nashville.

